# Economic surplus implications of Mexico’s decision to phaseout genetically modified maize imports

**DOI:** 10.1080/21645698.2021.2020028

**Published:** 2022-02-28

**Authors:** Diego Maximiliano Macall, William A. Kerr, Stuart J. Smyth

**Affiliations:** Department of Agricultural & Resource Economics, University of Saskatchewan, Saskatchewan, Canada

**Keywords:** Agroecology, financial loss, food prices, food security, glyphosate, trade

## Abstract

The Mexican government has decided to ban imports of genetically modified (GM) maize, to rely on agroecology for maize production to satisfy domestic yellow maize requirements. No economic impact assessment of this policy decision was made public, and the implications of this decision for users of yellow maize and consumers are significant. This article measures the economic surplus generated from Mexican GM yellow maize imports and domestic conventional yellow maize production over the last 20 years, and projects the economic surplus generated over five years from adopting agroecology for yellow maize production. We explore three likely scenarios and find that in all of them, yellow maize processors lose almost twice as much economic surplus as producers. In the most conservative loss estimate (Scenario 1), the surplus loss in five years is equivalent to 35% of the economic surplus generated over the last 21 years from GM maize imports and domestic Mexican conventional production. In all simulated Scenarios, between 2024 and 2025 the price of a metric ton of yellow maize will increase 81percent because of the change in production systems (from conventional to agroecology). These financial losses will ultimately factor into the prices consumers pay for poultry and red meat products, resulting in higher domestic retail food prices.

## Introduction

On 9 December 2020, a draft Decree calling for the phase-out of both glyphosate and genetically modified (GM) maize was made public in Mexico.^1^ Mexican President Andrés Manuel López Obrador stated that he was taking this decision because, “[w]e will not allow our people to be poisoned. It is not production for the sake of production, rather that food consumption adjusts to health recommendations.”^2,^^a^^a^Original Quote: “No vamos a permitir que se envenene a nuestro pueblo. No es producir por producir, sino procurar que el consumo de alimentos se ajuste a las recomendaciones sanitarias”. In text English translation by D. M. Macall.On 31 December 2020, the draft became a Presidential Decree ^3^^b^^b^See unofficial English translation at: https://www.fas.usda.gov/data/mexico-mexico-publishes-decree-ban-glyphosate-and-ge-corn. stating that the use, acquisition, distribution, promotion or import of glyphosate by public institutions would be banned in Mexico. The Decree instructs the Secretariats of Agriculture and Rural Development (SADR) and Environment and Natural Resources (SMARN) to promote and implement sustainable and culturally appropriate alternatives to glyphosate use such as: low toxicity agrochemical use; biological or organic product use; agroecological practices^c^^c^Agroecological practices, in addition to agricultural production, focus on the ecological sustainability of the production system. The Mesoamerican *milpa* system is an iconic example of this type of production system.; or the intensive use of labor. By no later than the first semester of 2023, the use of glyphosate will not be permitted in Mexico.^3^

Article 5 further states that by the first half of 2023, CONACYT^d^^d^Consejo Nacional de Ciencia y Tecnología (CONACYT) is Mexico’s entity in charge of the promotion of scientific and technological activities. It sets government policies for these matters. “ … will promote the reforms of the applicable legal systems to avoid the use of … genetically modified maize in Mexico.”^3^ There is no government approved commercial cultivation of GM maize in Mexico. Hence, it is not clear whether the Decree refers to GM maize for direct human consumption (white maize), or whether it refers to maize used in the commercial production of livestock feeds, whose primary ingredient is yellow maize. Article 6 of the Decree states that biosafety authorities will revoke and refrain from granting permits for the environmental release of GM maize seed. Article 6 has three aims, the first is to contribute to food security, second to contribute to food sovereignty, and third to act as a special measure to protect native maize cropping systems (*Milpa*), biocultural wealth, peasant communities, Mexican gastronomic heritage and the health of women and men. Likewise, biosafety authorities will revoke and refrain from granting authorizations for the use of GM maize in the diet of Mexican women and men, until it is fully replaced on a date no later than 31 January 2024, in accordance with food self-sufficiency policies.

The Mexican livestock feed sector voiced concerns about the Decree almost immediately after it was made public, indicating that they understand the Decree to mean that imports of GM maize will be banned from 2024 onwards.^4^ Juan Cortina, president of the Mexican National Farm Council, when referring to the perceived transgenic yellow maize import ban stated that: “Even if we wanted to import it from somewhere else, it does not exist, transgenic or non-transgenic. Those volumes do not exist so that Mexico could import them from somewhere else, especially in an efficient manner as it is currently acquired from the United States..”^5,^^e^^e^Original quote in Spanish: “Aunque quisiéramos importarlo de otro lado, no lo existe, transgénico o no transgénico. No hay esos volúmenes para que México pueda importar y mucho menos de la manera tan eficiente que hoy en día se tiene con Estados Unidos.” In text English translation by D. M. Macall. Almost all yellow maize used in Mexico is imported from the US, where the acceptance of GM maize approaches one hundred percent.^6,7^ After having discussed the issue with their Mexican counterparts, US agricultural authorities stated that they believed the import ban would only affect white maize (for human consumption), and not yellow maize.^8,9^ However, Mexican authorities have clarified their policy decision, maintaining that GM maize imports will be completely stopped by 2024.^8^ Their rationale is that the presence of GM maize at any point of the supply chain leading up to food products that will be consumed by Mexicans, is undesirable.

In Mexico, GM crop regulation is articulated by the *Biosafety Law of Genetically Modified Organisms* and is supposed to be carried out on a case-by-case basis. The law, which was enacted in 2005, outlines the mechanisms by which genetically modified organisms (GMOs) are to be imported, as well as the biosafety protocols they are subject to in case of their field release. Article 2, Section XI of the law details the precautions to be followed for certain crops, particularly those for which Mexico is the place of origin. Currently, 59 phenotypically distinct maize races have been described in Mexico; their genotypic differentiation is mainly due to isolation by distance.^10^ Within its biosafety law, Mexico has explicitly enshrined the protection of these maize landraces. However, controversy notwithstanding,^11^ this wealth of maize diversity has not deterred Mexican agricultural authorities from exploring the options GM maize offers.^12^

Between 1998 and 2005, 344 authorized GMO field trials were approved and conducted in Mexico.^13^ Between 2005 and 2019, under the authority of the *Biosafety Law of Genetically Modified Organisms*, 671 legal permits for the field release of eight distinct GM crops were granted. Most of these permits were for GM cotton (359), and the second largest were for GM maize (202).^14^ Moreover, *Bacillus thuringiensis* (Bt) and herbicide tolerant (HT) cotton varieties have been cultivated in Mexico since 1996. Prior to the introduction of these GM varieties, severe pest pressure made the cultivation of conventional cotton unfeasible.^15^ An early assessment of the technology under irrigation in the Comarca Lagunera region, noted a marked reduction in pesticide use (none needed for control of bollworm in Bt cotton, 2% of 1988 amounts for conventional cotton), and an overall positive experience with Bt cotton.^16^ A more recent assessment noted that Bt cotton has been responsible for the sustained decrease in pesticide use (one application in Sonora and Mexicali, five applications in La Laguna), and thus far, no cases of weed resistance to glyphosate associated with GM cotton have been reported in Mexico.^17^ However, GM cotton cultivation in Mexico has not been without controversy, as transgene introgression into wild cotton races has recently been detected.^18^

Mexico has a biotechnology regulatory regime that may ask for more than 100 different requirements to grant an experimental GM crop planting permit,^11^ but does allow the import of, and experimentation with, GM crops. No economic impact assessment of the decision to ban GM maize imports has been made public, if one was even undertaken. Such analysis would inform stakeholders (government, industry, farmers, and consumers) about the economic implications of this policy. This is particularly important given that according to CONAFAB ^6^: “Almost a quarter of all commercial feed manufactured in 2021 will be for broilers.” Another 14% of manufactured feed will be for layers, and another 18.4% will be for dairy cattle. In other words, 57% of total feed produced in Mexico is used to secure the production of three farm animals, from which chicken meat and eggs, as well as cheese, milk and yogurt (among other dairy products] are derived. These final products are consumed daily by millions of Mexican citizens; any price change to them will have effects on the overall food and nutritional security of Mexican society. This article analyzes the economic implications of Mexico’s decision to ban US GM yellow maize imports, utilizing a two-step process. First, a partial equilibrium framework is used to determine the economic surplus generated by yellow maize trade between Mexico and the US over the last 20 years. Second, the economic surplus method is used to project the change from Mexico’s decision to substitute conventional yellow maize production with agroecological maize production methods. This two-step approach allows for a better understanding of the implications of Mexico’s decision to ban GM maize imports and adopt agroecology maize production practices, by showing what the former policy has provided, and what the new policy offers into the near future.

## Maize Production in Mexico

Maize has been cultivated in North America for some 7,000 to 10,000 years; Mexico is considered the place of origin of the plant.^19^ In Mexico, maize is considered not simply another crop, it is perceived as an integral part of national identity and the local diet, especially by indigenous groups.^20^ In the Americas, Mexico has the highest per capita consumption of maize at 267 g/person/day^21^, and 34% of agricultural land is devoted to its cultivation.^22^ Traditionally, maize in Mexico has been produced in cropping systems called *Milpa*.^23^ In these production systems, farmers play a wide range of roles, they conserve genetic resources, select and plant seed saved from their crops, and after harvest, they are the main consumers of their products.

When, Quist and Chapela^24^ reported that they had detected transgenes (genes of a GM hybrid) in local maize varieties in the mountains of the state of Oaxaca, fear of losing genetic purity was stoked within Mexican society (especially among indigenous groups).^25^ However, Quist and Chapela’s results were immediately contested due to the methodology they used,^26^ and while the journal in which their work was published did not retract the article, it made readers aware of the controversy surrounding their results.^27^ Three years after that article, Ortiz-García et al.^28^ did a similar analysis but failed to detect any transgenes in local maize landraces. Regardless, Quist and Chapela’s study helped shape negative perceptions about GM maize in Mexico, which have had a lasting effect.^11^ Since the Quist and Chapela article, Mexican government sponsored scientific studies have detected transgenes in maize landraces, but no peer-reviewed summaries of this work have been published.^29^ Moreover, according to a Commission for Environmental Cooperation (CEC) assessment, whole grain GM maize enters Mexico through imports, but it may also be carried by migrant workers returning from the US.^29^ Mexican farmers sometimes plant these GM maize seeds to increase their own production.

**Figure 1.**
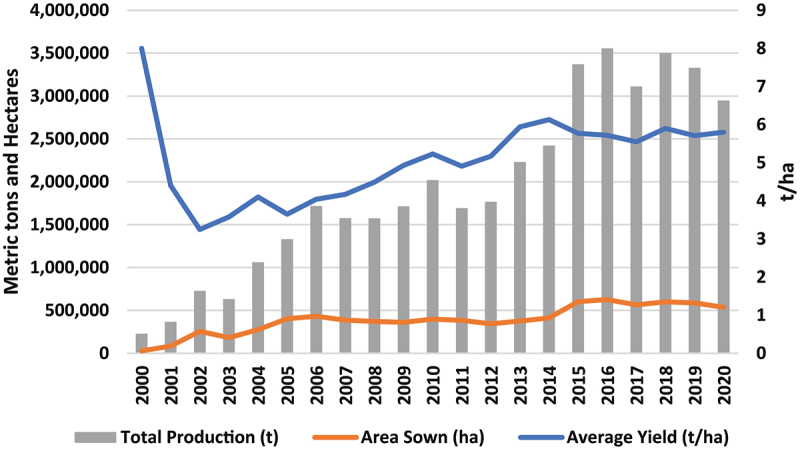
Mexican yellow maize production, area sown, and average yield, 2000–2020.

Source: Authors based on SIAP.^30^

Yellow maize in Mexico is produced under irrigation and through rainfed agriculture. This crop, in either production modality, is grown in 21 of the 32 Mexican states. Given Mexico’s diversity of soil types, altitudes, and topographies there is substantial heterogeneity in the types of yellow maize production systems throughout the country.^22^ Considering 2000 as the base year, between 2000 and 2020 there has been a 2,000% increase in the yellow maize area sown, and a 1,460% increase in total production (Fig. 1). Between 2003 and 2004, there was a significant production increase of 68%, which might be partly explained by Mexican migrant workers introducing and planting GM maize seed.^29^ Production dramatically increased after 2003, and the rise in yield cannot be solely explained by the release of yield enhancing domestic varieties.

Average yellow maize yield per hectare (t/ha) between 2000 and 2020 is 5 t/ha. Practically all yellow maize produced in Mexico, or imported from the US, is destined to livestock feed production.^6,31^

On average 384,000 ha are planted annually to yellow maize in the country. At 20-year average yield levels (5 t/ha) under a conventional production system (agrochemical use) if GM maize imports are banned, to meet current domestic demand, hectares planted to yellow maize in Mexico will have to increase by 512% (or to 2.35 M ha). However, the Presidential Decree calls for the adoption of agroecological practices, and the use of more intensive labor as the means of weed control, in Mexican agriculture.

Agroecology is an ambiguous term that could mean many different things depending on the context and stakeholders involved.^32^ Generally, the term refers to agricultural production without the use of any synthetic inputs. Organic farming, a production system that sustains agricultural production by avoiding the use of synthetic fertilizers and pesticides, is a production system that fits within the concept of agroecology.^32^ In Mexico, there are no official statistics on organic maize production; in the US, organic maize yield per hectare is on average 31% less than that of conventional maize.^33^ Taking the US organic maize yield as a proxy, a 31% reduction in yield per hectare in Mexico would result in an average yield of 3.45 t/ha. If Mexico adopts agroecological methods to supply its yellow maize demand, a total of 3.95 million hectares (761% increase over current number) would need to be annually planted with yellow maize.

**Figure 2.**
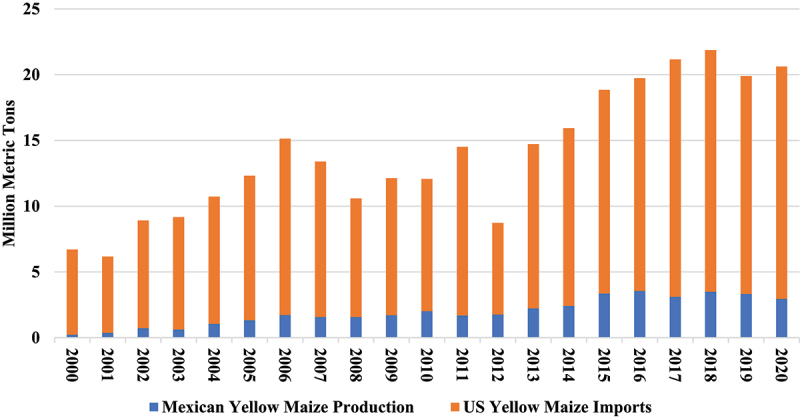
Mexican yellow maize production & imports, 2000–2020.

Source: Authors based on data from SIAP^30^, and ERS^7^.

Through conventional yellow maize production systems, between 2000 and 2020, Mexico produced only 14% of its annual yellow maize requirements (Fig. 2). The remaining 86% of yellow maize consumed over this time period has been imported from the US. It is evident that banning GM maize imports will have a profound impact on domestic livestock feed production, which in turn will influence the domestic price of end-consumer poultry and dairy products.

## Mexico, United States, and Canada Free Trade Agreement (T-MEC)

Mexico is a member of 13 free trade agreements (FTAs) with 50 countries, 29 agreements for the mutual promotion and protection of investments with 30 countries, and 9 limited reach agreements within the scope of the Latin American Integration Association.^34^ By deciding to phaseout GM maize imports, the trade relationship with the US will be directly affected. With the US, Mexico has a FTA called T-MEC (Spanish acronym) which also includes Canada, and is an updated version of the previous North American Free Trade Agreement.

According to Article 3.10, Part 1 of the Agricultural Chapter of the T-MEC, Mexico will have to inform the US of its intention to stop the import of GM maize because one of the reasons cited for this decision (in the Presidential Decree), is the suggestion that GM maize adversely impacts human health.^35,36^ This is in direct contradiction of the 4,485 risk assessments of GM crops, which included risk assessment for human health, conducted by national regulatory agencies in 72 countries, all of which concluded that the risks from GM crops are no different to the risks from the production of non-GM crops and foods.^37^ When T-MEC came into force, a Working Group (Article 3.16) for information exchange and cooperation on policy and trade related matters associated with products of agricultural biotechnology was created. This Working Group was tasked with providing a forum through which science-based information about GM crops can be exchanged. This mechanism could be used to discuss the various direct and indirect health, economic, and environmental benefits that GM crops have been shown to provide.^38–41^ In the specific case of GM maize, insect resistant varieties have shown to have 30% lower concentration of aflatoxins than conventional maize varieties.^42^ Maize aflatoxins are linked to increased rates of liver and esophageal cancers. Moreover, another mandate of the Working Group is to “advance regulatory approaches and trade policies that are transparent, and based on science and on risk for products of agricultural biotechnology in other countries and in international organizations.” Suggesting that GM crops have adverse effects on human health is contradictory to the consensus found in the scientific literature, which deems them no riskier than conventional crops. The US has legal recourse to launch a complaint against Mexico’s unfounded decision to block GM maize imports.

## Methodology

### Trade Policy Analysis

The workhorse of trade policy analysis is partial equilibrium, comparative static analysis.^43^ While this framework does not encompass general equilibrium interactions, nor does it provide dynamic paths of adjustment, it does offer the flexibility needed to assess complex markets and a wide variety of institutional arrangements that act to inhibit or enhance international trade. Additionally, the data requirements to construct such a framework are modest when compared to others.

**Figure 3.**
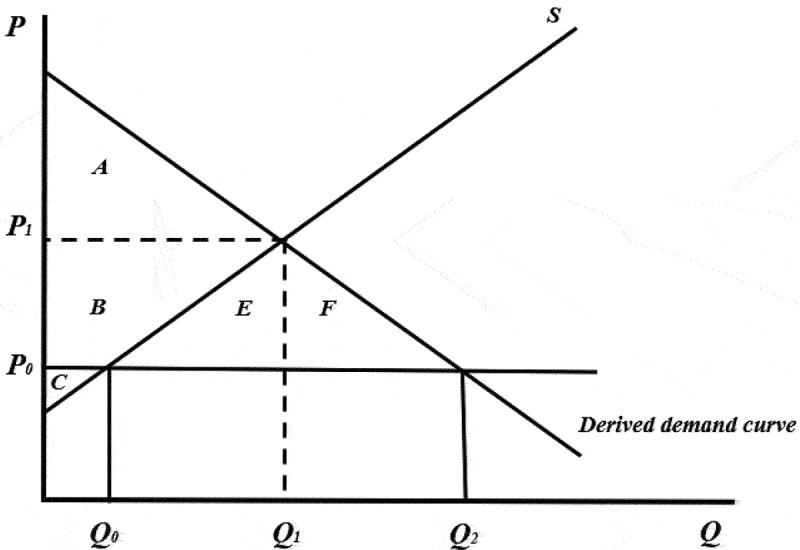
Mexican yellow maize market.

This framework was adapted and used to determine the economic surplus generated from both domestic production, and imports from the US, in the Mexican yellow maize market between 2000–2020 (Fig. 3). In Fig. 3 the demand curve shown is the derived demand from the livestock feed industry in Mexico.^f^^f^And not the final consumer demand for livestock products. As depicted in Fig. 3, both domestic production and yellow maize imports generate a total economic surplus equal to *A* + *B* + *C* + *E* + *F*. Economic surplus from imports and domestic production is calculated over this 20-year period. Mexican yellow maize supply and demand schedules were built with the elasticities of supply (0.22) and demand [−0.12) estimated for Mexico by FAPRI and used by Macall and Smyth ^44,^^g^^g^These elasticity values used in this study were estimated for maize in Mexico when FAPRI was still linked to Iowa State University. FAPRI is now part of the University of Missouri and no longer publishes elasticity values for commodities other than those produced and consumed in the US. and the average quantity of yellow maize produced, demanded and imported between 2000 and 2020. Over this 20-year period, the average farm-gate price of one metric ton of yellow maize in Mexico was US$191.

Currently, the USDA measures trade between the US and Mexico at the 2010 nominal exchange rate between the US dollar and the Mexican peso. Therefore, this exchange rate was used to determine the prices at which yellow maize was imported into Mexico over the last 20 years.^45^ A discount rate of 5% was applied to the net present value [NPV) measure of welfare as suggested by Ray^46^ Data to populate the framework were obtained from Mexico’s Agricultural Statistics Service (SIAP], and from the US Department of Agriculture’s Economic Research Service (ERS).^7,47^

Blocking yellow maize imports from the US would cause a loss of economic surplus equal to *E* + *F*, and the domestic yellow maize price would increase from *P_0_* to *P_1_* given that supply and demand would be governed by internal market forces (closed economy). However, it is unlikely that the domestic yellow maize agroindustry sector would go without the main ingredient for its business operations for very long. Moreover, the Mexican government is working so that current conventional agricultural practices can be substituted by practices akin to organic agriculture (i.e. no use of synthetic fertilizers or pesticides). Thus, it is illustrative to project the implications this policy would have on the yellow maize market as well.

Using the International Food Policy Research Institute’s DREAMpy software^48^^h^^h^DREAMpy is an open-source software that allows users to evaluate the economic impacts of agricultural research. The software is based on a flexible partial equilibrium model whose key variables can be manipulated, and the software also allows users to run sensitivity analyses on key variables and simulate a wide range of scenarios. the economic surplus of Mexico’s decision to change production systems from conventional maize production to agroecological production methods was simulated (see Annex 1).

**Figure 4.**
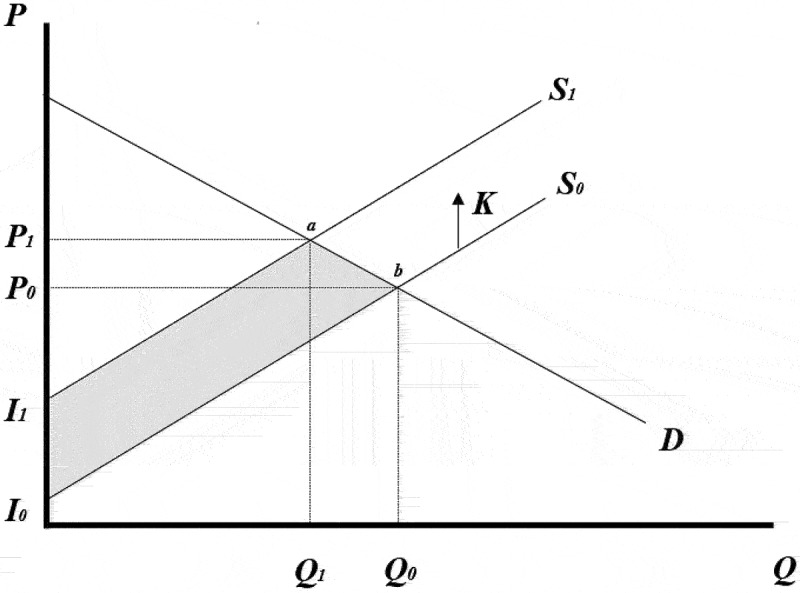
Mexican decision to implement an agroecological maize production system.

Mexico’s decision to adopt agroecology in lieu of conventional maize production can be understood as a technology rejection that will have effects on domestic yellow maize supply (Fig. 4). Beginning at an initial price and quantity equilibrium in the Mexican yellow maize market, agroecological maize is expected to decrease per hectare productivity. A productivity reduction is likely because agroecological production systems typically rely on ecological processes to manage biotic and abiotic stresses, instead of relying on inputs (synthetic or not). If yellow maize is grown under this production regime, it will be done on Mexico’s eroded soils,^49^ and under the attack of leaf-eating insects, which have been shown to reduce conventional maize yield in Mexico by between 13 and 17%.^50^ In other words, yellow maize plants in an agroecology production system are likely to go undernourished due to the restricted supply of nutrients through fertilizers and be under the attack of pests which will not be managed with pesticides. Thus, the domestic yellow maize supply curve shifts upward from *S_0_* to *S_1_*; whereas, derived demand for maize is assumed to remain unchanged. Linear curves and a parallel shift in supply (*K*) were assumed in order to model the impact of agroecological maize adoption.^51^ The price of yellow maize will increase from *P_0_* to *P_1_* because of expected supply reductions and decreased volume of maize produced. As a result, the economic surplus accruing to livestock feed producers deceases equal to the area *P_1_abP_0_*, the change in producer surplus is equal to the area *P_0_bI_0_* + *P_1_abP_0_*, and total surplus decreases equal to the area *I_0_baI_1_*. Calculations are computed for all the years of the consideration period (5 years) in which supply curve shifts are expected to be caused by agroecological maize production adoption. A consideration period of 5 years is used to build simulated scenarios because agroecology yellow maize production will transform Mexican agriculture and have market effects beyond yellow maize processors. After the initial shock, it is unlikely that five years on the Mexican yellow maize processor industry will be structured the same way as before maize was produced through agroecological methods. It is also unlikely the sector will be the same size as smaller and less efficient processors, who are unable to absorb new costs or pass them on to consumers, will likely exit the industry.

Three scenarios were simulated to capture a range of potential outcomes from Mexico’s decision to ban yellow GM maize imports and become self-sufficient in production of this crop, through an agroecological production system. Most yellow maize production in the country occurs in the state of Chihuahua, which borders the US states of New Mexico and Texas. The cost structure Mexican agricultural authorities have documented for this state was used for all economic surplus simulations (Table 1). The cost structures are assumed to be the same except for the fertilization component, which is assumed to not occur for agroecologically grown maize.^i^^i^Fertilization with organic matter may occur, but this activity can be ascribed to plot management in an agroecological production system. Plot management refers to all the activities farmers do [soil preparation, weeding, covering their crop with organic matter, etc.) throughout the lifecycle of the maize crop in order to guarantee maize production. Pest management in a conventional yellow maize production system is done through chemical means; in an agroecological production system, this activity will have to be carried out through the use of “intensive” labor.^j^^j^Pest management means the control of pernicious insects affecting yellow maize as well as weeds. According to Krishna,^53^ p. 134 “in order to obtain a decent maize crop a minimum of 40–60 weed-free days are essential.” Thus, the agroecologically grown yellow maize cost structure used to project surplus generated from this technology accounts for the additional labor needed to attain this requirement. An average number of 50 days were priced into the cost structure. The 2021 Mexican minimum per day salary that can be paid to an agricultural worker of $160.19 Mexican pesos (2010 nominal US$8.31), was used to estimate how much it would cost per hectare to guarantee maize production.^54^ Rather than add it to the US$217 needed to manage pests on a conventional yellow maize hectare, the 50 manual-weeding days required are substituted in the agroecologically-grown yellow maize hectare (labor’s economies of scope).

**Table 1.** Mexican yellow maize production cost structure.

Cost component	Conventional yellow maize	Agroecological yellow maize
Land Preparation	$154	$154
Planting	$217	$217
Fertilization	$407	$0
Plot management	$118	$118
Irrigation	$610	$610
Pest management [insects, weeds, diseases)	$217	$415
Harvest	$133	$92
Incidentals	$129	$129
**Cost per MT**	**$1,985**	**$1,735**

Source: Adapted from FIRA.^52^

Note: Prices are in USD at 2010 nominal USD/Peso exchange rate.

Given that agroecology yellow maize production will yield less per hectare, less labor will be needed for harvest under this production system. Brookes and Dinh^55^ note the significant difference in the amount of time spent harvesting a GM maize variety due to it having a higher yield, when compared to a conventional one that produces less (requiring less labor for harvest). Measured in dollars per hour, this labor-time difference affects the cost structure. For simulated scenarios in this study, given agroecology’s average −31% yield difference with conventional yellow maize production, it is assumed that the harvest cost component of agroecological yellow maize production proportionally decreases 31%.

**Figure 5.**
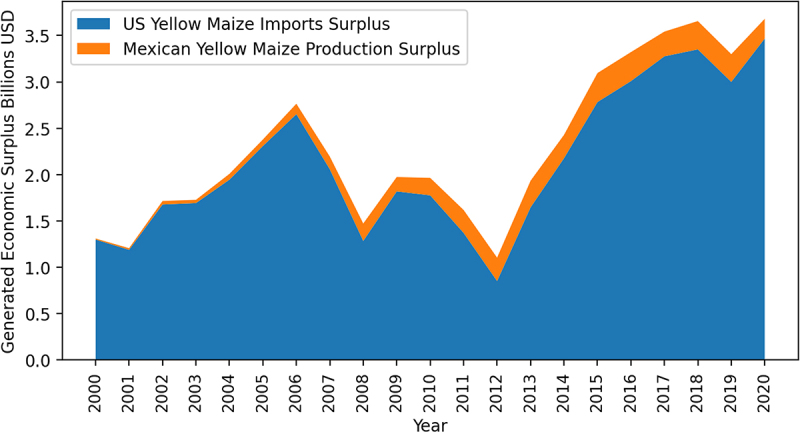
Mexican yellow maize production and imports surplus (2000–2019].

Note: A 5% discount rate to the NPV was applied to all surplus measures.

A summary of the assumptions made for each of the three scenarios simulated can be found in Table 2. The first Scenario is built on 20-year averages of key Mexican yellow maize production parameters. Scenario 3 is built on the latest year for which there is information (2020), as yellow maize production and imports have increased significantly since 2012 (Fig. 5). Scenarios 1 and 3 are static, that is, there is no change in demand or supply over the consideration period. Scenario 2 introduces a dynamic 1% year-on-year increase in yellow maize demand that corresponds to the average population growth Mexico has experienced over the last five years.^56^ For all scenarios it is assumed that agroecologically-grown yellow maize production will decrease yield by 31% as detailed above. The Presidential Decree states that pertinent Mexican authorities will work between 2021 and 2024 with Mexican yellow maize producers to teach them agroecological maize production methods, that is why it is assumed that 100% of them will adopt agroecology production practices from 2025 onwards and there will be no time lag to full adoption.

**Table 2.** Assumptions of parameters used.

Parameter	Scenario 1	Scenario 2	Scenario 3
Initial equilibrium price	191	191	191
Agroecology maize seed price	0	0	0
Equilibrium quantity metric tonne	13,636,908	13,636,908	20,612,784
Change in demand per year (%]	0	1	0
Current yield (t/ha)	5	5	5
Yield decrease (%)^a^	−31	−31	−31
Cost reduction (%)	12.6	12.6	12.6
Supply elasticity (*ε)*	0.22	0.22	0.22
Demand elasticity, absolute value (*η*)	0.12	0.12	0.12
Initial adoption level (%)	100	100	100
Maximum adoption level (%)	100	100	100
Lag to maximum adoption level [years)	0	0	0

a: In the US organic maize yields on average −31% per hectare compared to conventional maize. As no published statistics on organic maize production in Mexico were found, the value observed in the US as reported by NASS^33^ was used as a proxy for the yield that could be observed in Mexico if they produced yellow maize organically.

## Results & Discussion

### Trade Analysis Results

The total economic surplus generated over 20 years of yellow maize production and imports in Mexico is US$48.4 billion (Fig. 5). Most of the surplus generated comes from Mexican imports of yellow maize from the US. In other words, most of the surplus generated has benefitted the livestock feed sector which processes yellow maize in Mexico. This surplus benefits end-consumers in the form of more affordable poultry, bovine, and porcine food products. Since 2012, there has been a marked increase in both imports and domestic yellow maize production generating a greater amount of economic surplus. Had the import ban taken effect in 2020, a surplus loss of US$310 million would have been realized (loss of *E + F* in Fig. 3).

### Surplus Results

**Figure 6.**
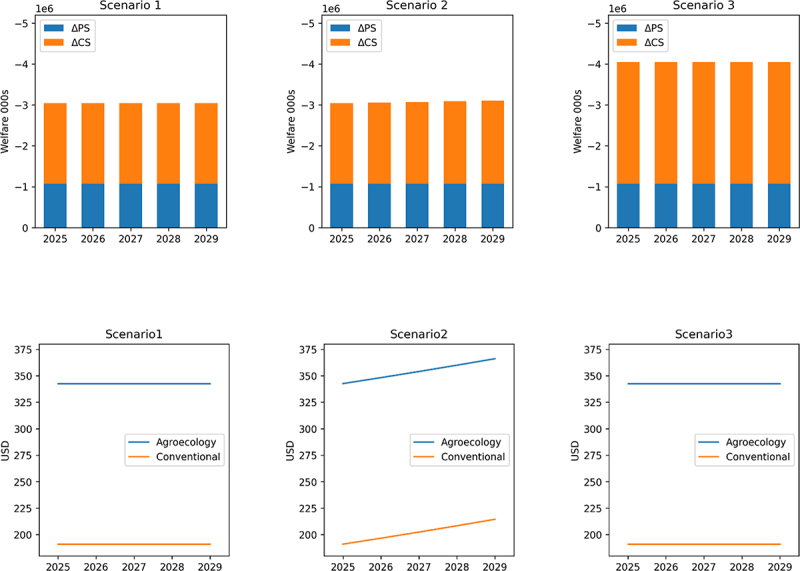
(a) Economic surplus from agroecology adoption results. (b) Equilibrium price per ton of yellow maize development across scenarios.

In all simulated scenarios, yellow maize processors lose more than Mexican yellow maize producers (Fig. 6a). This loss distribution is explained by supply being more elastic than demand (ε = 0.22, and *η = −0*.12), this relation makes the yellow maize processor’s share of losses due to yellow maize produced through agroecological methods, larger than the producer loss in all scenarios across all simulation years. In all three scenarios, for each year simulated (2025–2029), the producer share of total economic surplus loss is 35%, and the yellow maize processor share of the loss is 65%. In addition, the price per ton of yellow maize increases by 81% from one year to the next, a price shock that is sure to influence the prices of the main ingredient in the production of animal protein sources in Mexico (Fig. 6b). In Scenario 1, because no dynamism is introduced the simulation losses are constant over the period. Between 2025 and 2029, under the assumptions of this scenario, each year producers will lose US$1.07 billion in surplus and yellow maize processors will lose US$1.9 billion. In Scenario 2 because yellow maize demand increases by 1% year-on-year, losses for both Mexican yellow maize producers and processors increase as well as the equilibrium (farm-gate) price per ton of yellow maize. Under the assumptions of this scenario, the initial producer loss of surplus (in 2025) is US$1.07 billion and increases to US$1.1 billion, yellow maize processor share of losses begins at US$1.9 billion and increases to US$2.03 billion. Also within this scenario, as demand grows by one percent annually during the entirety of the consideration period, the price per ton of yellow maize will rise from US$343 to US$366. This latter price represents a 92% increase above the 20-year average price per ton of yellow maize in Mexico (US$191). Yellow maize processors will have to adjust to this new price in just five years, after having initially adjusted to an 81% price shock. Lastly, in Scenario 3 which is built on data from 2020 and has no dynamic simulation elements, every year of the consideration period the total loss in economic surplus will be US$4.6 billion. Across all simulated scenarios, the total welfare loss the Mexican economy would experience is measured in the billions of dollars.

Mexico’s decision to ban GM maize imports and supply its internal yellow maize demand through agroecological maize production will be costly. In the first five years of this policy coming into force, in the most conservative loss estimate (Scenario 1) US$15.2 billion in economic surplus will be lost. In the scenario built on the latest data (Scenario 3), US$22.2 billion are lost within the first five years of the policy coming into force. If the adopted policy develops according to this Scenario, in a quarter of the time it took to generate US$48.4 billion in economic surplus from both domestic production and trade with the US, Mexico will lose the equivalent of 48% of this total loss of economic surplus.

The removal of glyphosate as an effective method of weed control, will result in an increase in hand weeding, a practice defined as drudgery.^57^ In their assessment of drudgery work regarding the adoption of GM maize in South Africa, Gouse et al., identified that women spent 10–12 fewer days hand weeding their maize fields per season, allowing them to have larger vegetable gardens as they had more time to haul water to irrigate their garden, contributing to improved household food security. The Mexican government has given no thought as to how the increased demand for field labor will be supplied as the vast majority of current laborers work in the seasonal production and harvest of fruits and vegetables in the US and Canada. To secure the additional labor needed for hand weeding, farmers will be forced to pay higher wages, reducing their profitability, or forgo hand weeding, which will also reduce yields and profitability.

## Conclusions

The decision to ban GM yellow maize imports effectively renders Mexico’s biotechnology regulatory regime and commitments made under T-MEC moot. The loss from this policy decision will be substantial assuming the government is able to completely substitute conventional yellow maize production with agroecological production methods in a mere three years. Any environmental benefits gained from the reduced use of agrochemicals thanks to this policy, will be dwarfed by the significant costs along the yellow maize supply chain. One of the tenets of Article 6 of the Presidential Decree claims that its aim is to contribute to food security. It is difficult to see how increasing the price of yellow maize in Mexico will enhance food security.

The increase in the price of the main ingredient of livestock feed in Mexico raises the question of its impact for end-consumers of livestock-derived food products. Jiménez-Rodríguez and Morales-Zumaquero^58^ measured the relation between commodity prices and producer/consumer prices and found that for a developing country the “pass-through” rate between commodity prices and consumer prices is low. However, in this case the price shock from one year to the next is an 81% increase in price per ton of yellow maize. It is unlikely that only yellow maize processors and producers will absorb this price increase, undoubtedly some of it will be passed on further down the supply chain to end-consumers of livestock-derived products.

As a palliative measure, yellow maize processors might substitute a portion of their main animal feed ingredient with other ingredients such as other cereals and crop derivatives like distillers’ grains or oil meals. However, given that each year on average yellow maize processors require 13.6 million tons of yellow maize, it will not be simple to find acceptable alternative sources to supply the entirety of the required quantity. Furthermore, it is unlikely Mexico will find enough foreign sources of non-GM or organic yellow maize to satisfy its domestic demand for this crop. Assuming Brazil or Argentina did produce enough non-GM or organic yellow maize to satisfy Mexican demand, exporting the commodity would imply it traversing thousands of kilometers making the final price per ton too high for Mexican processors. Central America is not a likely source of yellow maize either as most countries in this region are barely self-sufficient in maize production and combined do not have enough spare hectares to produce 13.6 million tons of yellow maize annually. Mexico’s woes are compounded by the short amount of time the industry has to restructure itself to adjust to an agroecology reality.

## Annex 1 Total Economic Surplus Generated from Yellow Maize Trade and Domestic Production

**Table:**

Year	Yellow maize imports from the US surplus	Domestic yellow maize producer surplus	Total economic surplus generated by US GM corn imports
2000	$1,364,700,492	$11,231,843	$1,310,411,748
2001	$1,248,737,841	$18,387,978	$1,206,786,494
2002	$1,761,373,703	$39,926,154	$1,715,523,674
2003	$1,777,780,248	$37,008,712	$1,728,370,438
2004	$2,043,034,761	$65,802,480	$2,008,416,421
2005	$2,423,847,891	$74,021,607	$2,378,923,332
2006	$2,784,870,306	$117,702,992	$2,764,355,522
2007	$2,160,978,064	$143,610,370	$2,194,846,128
2008	$1,349,423,328	$196,818,042	$1,472,610,828
2009	$1,909,935,150	$162,947,394	$1,974,173,851
2010	$1,865,377,051	$197,800,245	$1,964,930,758
2011	$1,440,043,694	$259,361,780	$1,618,481,404
2012	$895,114,730	$264,747,374	$1,104,630,576
2013	$1,728,902,148	$301,187,178	$1,933,418,406
2014	$2,285,888,153	$264,075,948	$2,428,537,239
2015	$2,921,596,815	$328,271,438	$3,095,112,622
2016	$3,158,626,721	$328,660,300	$3,321,225,734
2017	$3,439,425,393	$280,020,539	$3,542,329,459
2018	$3,517,896,050	$320,795,336	$3,655,896,558
2019	$3,150,151,315	$313,548,385	$3,298,761,619
2020	$3,641,145,292	$223,518,027	$3,680,631,732

**Notes**: Economic surplus in 2010 nominal USD. NPV discount rate of 5% applied to total economic surplus measures.

***Economic Surplus Change Analysis***Agroecology can be treated as a process which the Mexican government has decided that domestic maize production will adopt. Following Alston et al. (^59^1995) the annual change in producer surplus (*ΔPS*) and change in surplus under the derived demand curve (*ΔCS)* from the adoption of agroecology maize can be calculated as:

(1) $$\Delta PS=P_{t}Q_{t}\left(K_{t}-\;Z_{t}\right)\;(1\;+\;0.5Z_{t}\eta )$$

(2) $$\Delta CS=P_{t}Q_{t}Z_{t}(1\;+\;0.5Z_{t}\eta )$$

Where *P_t_* and *Q _t_* are the initial equilibrium price and quantity. *K_t_* is the parallel^11^ ^11^ A parallel shift in supply is assumed as there is no information on the shape of a supply curve based on agroecological production. shift in the supply curve in year *t* due to agroecology maize and is estimated as:

(3) $$K_{t}=\;\left\{\left[E\;\left(Y\right)\right]/\varepsilon \;-\;\left[E\left(C\right)\right]/\left[1\;+\;E\;\left(Y\right)\right]\right\}\;p\;A_{t}\left(1-\delta_{t}\right)$$

Where *E(Y)* is the expected proportionate yield change per hectare, *ε* is the price elasticity of supply, *E(C)* is the proportionate change in variable input costs per hectare to achieve the expected yield change, *p* is the success rate or the probability that agroecology maize will achieve the expected yield, *At* is the adoption rate, and *δ_t_* is the rate of annual depreciation of agroecology maize (reduction of expected yield) in year *t. p* is assigned a value of 1 because agroecology is used in Mexico. *δ* is assigned a value of 0 because no change in yield is anticipated during the consideration period (five years).

*Z* is the absolute value of the change in price as a result of the supply shift and can be calculated as:

(4) $$Z_{\;}{=}_{\;}K\varepsilon /\;\left(\varepsilon +\eta \right)$$

Where *η* is the absolute value of the price elasticity of demand.
